# Viral Production in Seawater Filtered Through 0.2-μm Pore-Size Filters: A Hidden Biogeochemical Cycle in a Neglected Realm

**DOI:** 10.3389/fmicb.2021.774849

**Published:** 2021-11-18

**Authors:** Yanhui Yang, Toshi Nagata

**Affiliations:** Atmosphere and Ocean Research Institute, The University of Tokyo, Kashiwa, Japan

**Keywords:** bacteria, carbon flux, dissolved organic matter, low nucleic acid content bacteria, marine environments, viral production, virus reduction assay

## Abstract

Viral production is a key parameter for assessing virus-mediated biogeochemical cycles. One widely used method for the determination of viral production, called the virus reduction assay, reduces viral abundance, while maintaining bacterial abundance, using 0.2-μm pore-size filters. Viral production is estimated from the increase of viral abundance during incubation. We hypothesized that small-cell-sized bacterial communities can pass through 0.2-μm filters and drive viral production, representing a missing fraction of viral production that is missed by the virus reduction assay. Coastal seawater was filtered through 0.2-μm filters and diluted with virus-free seawater. Viral production in the <0.2-μm filtrate was estimated from changes in viral abundance determined through flow cytometry. We found that viruses were produced in the <0.2-μm communities, which were strongly enriched with low nucleic acid content bacteria. Estimated viral production in the <0.2-μm filtrates accounted for up to 43% of total viral production and 10% of dissolved organic carbon production mediated by viral lysis of bacterial cells. By not considering viral production in these <0.2-μm communities, the virus reduction assay may underestimate viral production. Virus–bacteria interactions in <0.2-μm communities may represent a significant and overlooked role of viruses in marine food webs and carbon fluxes.

## Introduction

Viruses are major agents of bacterial mortality and play important roles in the regulation of biogeochemical cycles in marine environments (Weinbauer, 2004; Suttle, 2007). Viral infection of bacteria drives the conversion of biomass into dissolved organic matter (DOM), which is available for bacterial re-consumption. The resulting cyclic flows of carbon and other elements may enhance DOM remineralization and decrease the efficiency of trophic transfer (Fuhrman, 1999; Wilhelm and Suttle, 1999; Motegi et al., 2009), although this process may also facilitate the production of refractory DOM (Nagata and Kirchman, 2000; Jiao et al., 2010). Viral production is a key parameter for assessing the magnitude of viral-mediated biogeochemical cycling. Several methods have been proposed to determine viral production in aquatic environments (Helton et al., 2005; Winget et al., 2005; Weinbauer et al., 2010). One widely used method, called the virus reduction assay (Wilhelm et al., 2002; Winget et al., 2005; Weinbauer et al., 2010), reduces viral abundance relative to ambient levels while maintaining near *in situ* bacterial abundance through the use of 0.2-μm pore-size filters. The seawater samples with reduced viral levels are used for subsequent incubation. Because the reduction in viral abundance inhibits new infection, viral production can be estimated by counting viruses originating from the lysis of previously infected cells. Combined with flow cytometric counting of viruses (Brussaard, 2004), the virus reduction assay has been extensively used in investigations of the roles of viruses in the regulation of bacterial dynamics and biogeochemical cycles (Malits et al., 2014; Kostner et al., 2017; Lara et al., 2017; Vaque et al., 2017; Mojica et al., 2020; Weinbauer et al., 2020).

One problem with the virus reduction assay is a low bacterial recovery; about 10–50% of bacteria can be lost during the 0.2-μm filtration process using tangential flow filtration systems (Winget et al., 2005). Although this loss is corrected for through calculation of the bacterial recovery rate, it is a potential source of error in viral production estimates. This loss of bacteria may be partly attributed to the passage of small-cell-sized bacteria through 0.2-μm filters; roughly 10% of marine bacteria may pass through 0.2-μm filters, depending on the filter type, filtration method and condition, and environment sampled (Zimmermann, 1977; Li and Dickie, 1985). However, viral production mediated by bacterial communities in <0.2-μm filtrates and its potential effect on viral production estimation have not yet been elucidated.

Here, we hypothesized that small-cell-sized bacterial communities capable of passing through 0.2-μm filters mediate viral production, representing a fraction missed by the virus reduction assay. To test this hypothesis, coastal seawater samples were filtered through 0.2-μm filters and diluted with virus-free seawater. Viral production in the <0.2-μm filtrate was assessed based on changes in viral abundance determined through flow cytometry.

## Materials and Methods

### Seawater Sampling and Cleaning of Containers Used for Experiments

Seawater samples were collected in Otsuchi Bay, located on the Pacific coast of northeastern Japan (39°20′36″N, 141°56′47″E; water depth: 46 m) on January 24, March 11, May 30, and July 24 of 2013. Dynamics of bacteria and viruses in Otsuchi Bay have been described elsewhere (Nagata et al., In press). Samples were collected at a depth of 5 m using a 12-L Niskin bottle (Sea-Bird Scientific, United States), gravity filtered through a 20-μm mesh-size nylon, and then used for subsequent analysis. Prescreening was used to remove larger particles, including occasionally abundant diatom cells (Tachibana et al., 2017) that can clog filters. Bacteria associated with particles larger than 20 μm were not considered in this study. All sampling tanks, cylinders, and incubation bottles used for the experiments were thoroughly cleaned through sonicating in alkaline detergent (5% Extran, Merck Ltd., Germany) and then soaked overnight. Then, the containers were rinsed with hot water to remove the detergent, and then rinsed with Milli-Q water. After that, the containers were soaked in 1 N HCl overnight and thoroughly rinsed with Milli-Q water. Before each incubation experiment, the incubation bottles and cylinders were rinsed with virus-free seawater (see below) three times to wet their inner surfaces.

### Virus Reduction Assay

Viral production was measured according to the method of Winget et al. (2005). Approximately 1 l of seawater sample was concentrated using a 0.2-μm pore-size polyethersulfone tangential flow filtration filter (Vivaflow 200, Sartorius Stedim Lab Ltd., Germany). After the concentrate reached about 100–200 ml in volume, virus-free seawater (filtrate from 100-kDa polyethersulfone filter tangential flow filtration; Vivaflow 200, Sartorius Stedim Lab Ltd., Germany) was added to the original volume. This washing procedure was repeated one or two additional times. In the final reduction step, the sample volume was reduced to approximately 100 ml. The filter was then slowly back-flushed to recover the remaining concentrate in the filtration cartridge and tubes. Seawater was pumped through the tangential filtration system using a peristatic pump (Masterflex L/S variable speed modular drive 7553-80, Cole-Parmer, United States), with a gentle backpressure of approximately 20 kPa (2.9 psi). This backpressure is less than half of that used by Winget et al. (2005) with the recommended flow conditions (8–10 psi). The concentrate was reconstituted with virus-free seawater to its original volume, and then incubated in triplicate 125-ml polycarbonate bottles (Nalgene, Thermo Fisher Scientific, United States). Samples were incubated at *in situ* temperature in the dark. Subsamples of 2 ml were collected every 3 h for the first 12 h and then at 24 h. Subsamples were placed in cryovials (Nalgene, Thermo Fisher Scientific, United States) and fixed with 0.02-μm (Anotop™ 25, Whatman, United Kingdom) prefiltered glutaraldehyde (20% solution for electron microscopy, FUJIFILM Wako Pure Chemical Co. Japan), at a final concentration of 1%, for 15 min in the dark. The samples were flash frozen in liquid nitrogen and stored at −80°C until flow cytometric analysis (see below). The viral production rate was determined as the slope of the linear regression of viral abundance against time, with a correction for bacterial loss due to sample processing (Winget et al., 2005).

### Viral Production in <0.2-μm Filtrates

In theory, the virus reduction assay is applicable to <0.2-μm communities, using finer (e.g., 0.1-μm) pore-size filters. However, the filtration process would result in further loss of bacterial cells and, thus, may introduce analytical challenges in detecting viral production. Furthermore, a significant fraction of bacteria may pass through 0.1-μm pore-size filters (Fukuda et al., 1998). For these reasons, we did not perform the virus reduction assay using <0.2-μm communities. Instead, we examined the net increase of viral abundance in <0.2-μm communities in samples that were or were not diluted using virus-free seawater. With dilution, both bacterial and viral abundances are reduced by the same proportion. Because the encounter rate of cells and viruses is the product of bacterial abundance, viral abundance, and the encounter kernel (Brownian collisions in the case of <0.2-μm communities; Murray and Jackson, 1992), a dilution factor of ×0.5, for example, means that the encounter rate between cells and viruses is reduced to 0.25 (=0.5^2^) times its original value. Thus, viral re-infection during incubation is strongly inhibited by dilution. The increases of viral abundance in both undiluted and diluted samples indicate that viral production occurs due to previously infected cells. If viruses increase only in undiluted samples and not in diluted samples, viral production is likely to be attributable to re-infection. A disadvantage of the dilution approach is that, in contrast to the virus reduction assay, both bacterial and viral abundance decrease by the same proportion, complicating detection of viral production at high dilution rates. Therefore, we prepared a dilution series (except in July) and assumed that if viral abundance increased in at least one of the dilutions, viral production was associated with previously infected cells. Note that our approach differs from the dilution method used for determining the grazing pressure and virus-induced mortality of phytoplankton (Worden and Binder, 2003), in which a response of the apparent cell growth rate to the extent of dilution is analyzed to estimate cell mortality. In our case, we simply followed the idea of the virus reduction assay using a dilution series.

Dilution series were prepared by diluting the <0.2-μm filtrate with virus-free sea water (100-KDa filtrate, see description above) to 20, 40, and 70% of the initial concentration, or left undiluted (100%), hereafter referred to as the 0.2, 0.4, 0.7, and 1 dilutions (the values indicate the fraction of original seawater), respectively (in July, we prepared only the 0.2 dilution). Triplicate of 100-ml samples of the diluents were placed into125-ml Nalgene polycarbonate bottles and incubated at *in situ* temperature in the dark. Samples for flow cytometric analysis were collected at a 3-h interval over 12 h (referred to as T0, T3, T6, T9, and T12) and at 24 h after the beginning of the incubation (T24). Subsamples for flow cytometry were collected in cryovials (Nalgene, Thermo Fisher Scientific, United States) and fixed with 0.02-μm (Anotop™ 25, Whatman, United Kingdom) prefiltered glutaraldehyde (20% solution for electron microscopy, FUJIFILM Wako Pure Chemical Co., Japan), at a final concentration of 1% for 15 min in the dark. The samples were flash frozen in liquid nitrogen and stored at −80°C until further flow cytometric analysis (see below).

The time courses of viral abundance were discontinuous (see section “Results”). The difference between the maximum and minimum viral abundance (ΔVA) was calculated over the time interval during which viral abundance increased significantly. The frequency of infected cells (FIC) was estimated by dividing ΔVA by (BS × BA) (Winter et al., 2004), where BS is burst size and BA is bacterial abundance at T0. We assumed a BS of 24, the average obtained in marine environments (Parada et al., 2006, 2008). The sensitivity of FIC estimates to the assumed BS was examined using a range of BS values (20–40) (Winter et al., 2004).

Viral production in the <0.2-μm filtrate (VP_<0.2_) was estimated using a modification of the “piece-wise” method (Winter et al., 2004; Kostner et al., 2017). This method was proposed for determining viral production through the virus reduction assay in cases where viral abundance shows a discontinuous (pulsed) dynamic rather than a linear increase. Viral production is estimated by dividing the ΔVA or the sum of ΔVA by the time elapsed from the beginning of the incubation to observation of the last peak (Winter et al., 2004). In our modified method, we divided ΔVA by 1 (day), assuming a diel cycle of viral production (Winter et al., 2004; Aylward et al., 2017). Where applicable, VP_<0.2_ was derived through correction with the dilution factor. Our estimates of VP_<0.2_ are conservative because they are based on the net increase in viral abundance with no correction for viral decay. For one case where re-infection was suspected (T0–T3 in March, see section “Results”), the corresponding ΔVA value was excluded from the calculation of FIC and VP_<0.2_.

### Flow Cytometric Analysis of Bacteria and Viruses

Viruses and bacteria were counted using flow cytometry (Brussaard, 2004; Yang et al., 2010). Prior to analysis, samples were slowly thawed on ice. To count viruses, samples were diluted 100-fold with Tris-ethylenediaminetetraacetic acid (EDTA) buffer (hereafter, TE; 10 mM Tris-hydrogen chloride, 1 mM EDTA, pH 8.0; Nippon Gene Co., Japan; prefiltered through 0.02-μm pore-size inorganic membrane filters, AnotopTM 25; Whatman, United Kingdom). Then, the samples were stained with SYBR Green I (Molecular Probes, United States) at a final concentration of 5 × 10^–5^ at 80°C for 10 min at room temperature in the dark. To obtain bacterial counts, samples were diluted 10-fold with TE buffer, stained with ×10^–4^ SYBR Green I, and incubated for 15–30 min at room temperature in the dark. After the addition of 1-μm yellow-green beads (Molecular Probes, United States), samples were injected into the flow cytometer (FACSCalibur; Becton Dickinson, United States). For both viral and bacterial samples, three blanks (treated in the same manner but without the addition of seawater samples) were prepared alongside the samples. The optimal voltage range of the green fluorescence (FL1) photomultiplier was determined by checking the electronic noise level of 0.02-μm filtered TE buffer. Using this voltage setting, three blanks were run to identify a noise window for separation of viral or bacterial counts from noise. The contribution of noise to the total count was always <5% for viruses and <0.1% for bacteria. High and low nucleic acid content bacteria (HNA and LNA bacteria, respectively) were discriminated on the cytogram of side scatter versus FL1. Consistent with previous observations in diverse aquatic environments (Li et al., 1995; Bouvier et al., 2007), the HNA and LNA bacteria were clearly distinguishable on visual inspection of the cytogram. Therefore, manual gating was used to discriminate these subgroups (Bouvier et al., 2007). The flow cytometric data were analyzed using the CellQuest software (Becton Dickinson, United States).

### Statistical Analysis

SigmaPlot v.14.4 (Systat Software, United States) was used for statistical analysis. Whenever the viral abundance increased between two sampling times, the difference in viral abundance between each time interval was assessed using Student’s *t*-test (*p*-values < 0.05 were considered significant), except for between T0 and T3. Because we took only a single measurement at T0, the significance of the difference in viral abundance between T0 and T3 could not be tested using Student’s *t*-test, and instead we used the 95% confidence interval of the T3 value. If the T0 value does not overlap with the confidence interval of the T3 value, we considered the T0 value significantly different from the T3 value. For calculation of error propagation, the coefficient of variability (CV, standard deviation/mean × 100) of the T0 value was assumed to equal the CV of the T3 value. The CV values for viral abundance estimates for triplicate incubations were generally <5%.

## Results

### Environmental and Microbiological Conditions

Experiments were conducted in winter (January and March) and the stratified period (May and July) (Supplementary Table 1). Table 1 summarizes the bacterial and viral abundance parameters in the filtrate of the tangential flow filtration. This table is an overview, rather than an assessment, of the differences in parameters among months (because the tangential flow filtration, which uses expensive filter cartridges, was conducted only once for each month, the uncertainty of each parameter associated with the operational error was unknown). The fraction of LNA bacteria that passed through the 0.2-μm filter was 17–35%, with a corresponding value for HNA bacteria of 0.08–5%. The contribution of LNA bacteria to the total bacterial communities in the 0.2-μm filtrate was 78–99% (Table 1). The fraction of viruses that passed through the 0.2-μm filter was 68–89% (Table 1).

**TABLE 1:** Abundances and fractions of bacteria and viruses that passed through 0.2-μm filters.

	January	March	May	July
Bacterial abundance in <0.2-μm filtrate (×10^4^ cells ml^–1^)	8.3	5.8	17	45
% of bacteria passed through 0.2-μm filter	7.8	8.7	8.1	15.2
% of LNA bacteria passed through 0.2-μm filter	25.8	16.7	18.0	34.7
% of HNA bacteria passed through 0.2-μm filter	0.08	0.38	1.30	5.12
% of LNA bacteria in <0.2-μm community	99.2	97.9	90.4	77.7
Viral abundance in <0.2-μm filtrate (×10^6^ viruses ml^–1^)	5.6	6.1	18	23
% of viruses passed through 0.2-μm filter	68.4	84.5	89.3	79.2

### Virus Reduction Assays

Viral abundance increased linearly during the initial 6 or 12 h of incubation (*r*^2^ = 0.50–0.92; *p* < 0.05, Supplementary Figure 1). Viral production estimated using the virus reduction assay (VP_*red*_; 10^9^ viruses l^–1^ d^–1^) was lower in winter (3.41 ± 0.23 in January and 1.05 ± 0.34 in March) than during the stratified period (14.2 ± 0.55 in May and 8.36 ± 2.13 in July).

### Dynamics of Viruses in <0.2-μm Filtrates

**FIGURE 1:**
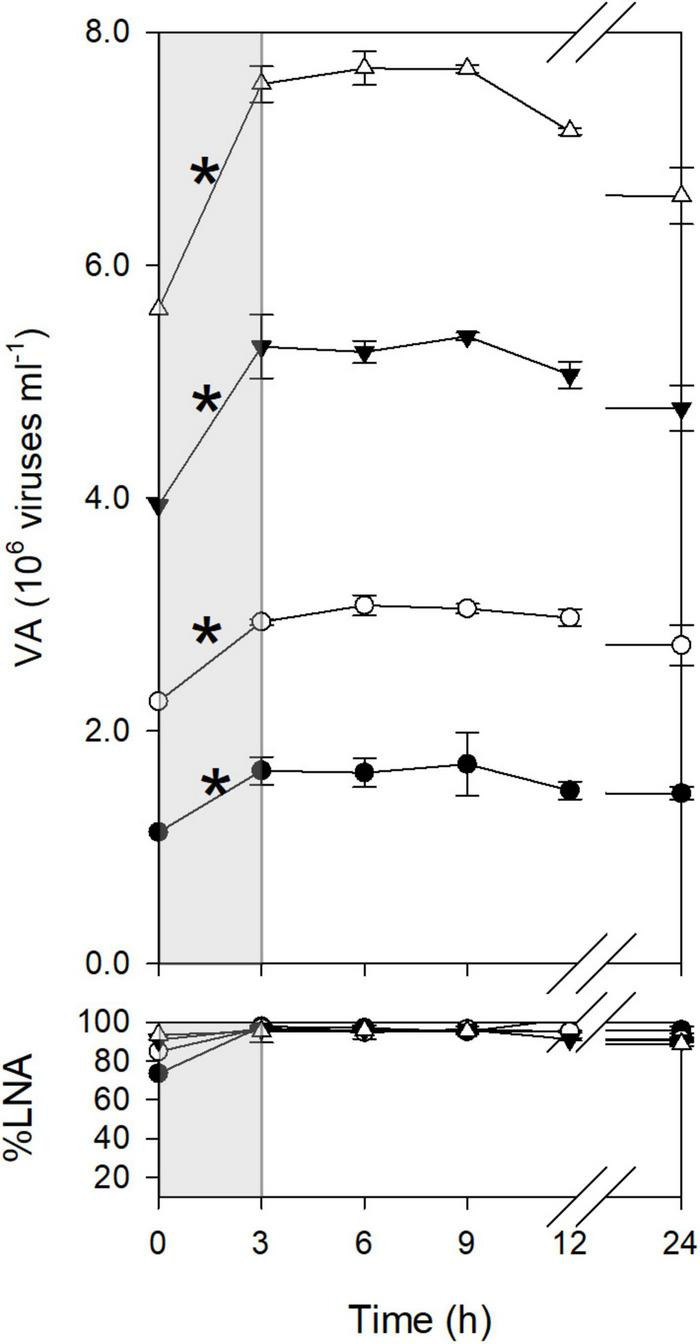
Dynamics of viruses during incubation (January experiment). The bottom panel shows the percentage of LNA bacteria to total bacterial abundance. Open triangles, 1 dilution; closed triangles, 0.7 dilution; open circles, 0.4 dilution; closed circles, 0.2 dilution. Errors are the standard deviations of the measurements using triplicate bottles (*n* = 3). The gray shading highlights a time interval with a significant increase in viral abundance, as indicated by asterisks. The significance of the difference between T0 and T3 was tested using the 95% confidence interval (see section “Materials and Methods”).

Viral dynamics in the <0.2-μm filtrates were discontinuous. For example, in January, viral abundance increased significantly between T0 and T3 (Figure 1). After T3, viral abundance remained constant until T9 and then declined. Similarly, in other months, viral abundance increased during only a certain time interval (Supplementary Figure 2). These increases in viral abundance were considered to reflect viral production from previously infected cells, as viral abundance increased in diluted samples. An exception was found for the time interval of T0–T3 in March, when viral abundance increased only in the undiluted treatment. The ΔVA for this time interval was not used for the calculation of FIC and VP_<0.2_ (see section “Materials and Methods”). LNA bacteria dominated the community (%LNA > 80%) throughout the incubation period except for the later portion of incubation in May and July, when %LNA decreased after T12 (Figure 1 and Supplementary Figure 2). We found no substantial decrease in %LNA during the time intervals when viral abundance increased.

### Frequency of Infected Cells and VP_<0.2_

The FIC was estimated using the data from the different dilutions in January, March, and May. The FIC estimates were consistent among different dilutions, except in March when the FIC for the 1 dilution was approximately half of those for the 0.4 and 0.7 dilutions (Table 2). The FIC was also sensitive to the BS assumption. Using the range of BS (20–40) reported by Winter et al. (2004), FIC estimates differed twofold depending on the BS assumption. For each month, the average FIC using the default BS setting (24) ranged from 42 to 93% in January, March, and May, and was 7% in July (Table 2). The average VP_<0.2_ (10^9^ viruses l^–1^ d^–1^) for each month varied in the range from 0.76 to 3.99 (Table 3). From VP_<0.2_ and VP_*red*_, we estimated the total viral production, VP_*tot*_, using the following equation: VP_*tot*_ = (1−*f*) × VP_*red*_ + VP_<0.2_, where *f* is the fraction of bacteria that passed through the 0.2-μm pore-size filter. VP_*red*_ is multiplied by (1−*f*) because the calculation of VP_*red*_ corrects for any loss of bacteria during the tangential flow filtration using the bacterial recovery rate (this correction implicitly includes the loss of bacteria due to the passage of bacteria through 0.2-μm pore size filters). Then we examined the percentage of VP_<0.2_ relative to VP_*tot*_ and found that VP_<0.2_ accounted for a large proportion (36–43% on average) of VP_*tot*_ in January, March, and July, although the percentage of VP_<0.2_ in May was lower (11%) (Table 3). Comparison of the VP_*red*_ and VP_*tot*_ revealed that VP_*tot*_ was 1.3–1.7 times higher than VP_*red*_ in January, March, and July, whereas VP_*tot*_/VP_*red*_ was near 1 in May (Table 3).

**TABLE 2:** FIC of <0.2-μm communities.

	Dilution	FIC
January	0.2	104 ± 24 (62–125)
	0.4	78 ± 3 (47–93)
	0.7	95 ± 19 (57–114)
	1	97 ± 13 (58–116)

	**Mean**	**93**

March	0.4	81 ± 25 (49–98)
	0.7	85 ± 21 (51–103)
	1	40 ± 11 (24–48)

	**Mean**	**67**

May	0.7	37 ± 8 (22–45)
	1	47 ± 22 (28–57)

	**Mean**	**42**

July	0.2	7 ± 2 (4–8)

*Errors are standard deviations (n = 3; with error propagation for the measurement using triplicate bottles). Values in parentheses are FIC estimates obtained using different BS assumptions ranging from 20 to 40 (see text for explanation).*

**TABLE 3:** Comparison among VP in the <0.2-μm filtrate (VP_<0.2_), VP estimated from the virus reduction assay (VP_*red*_), and total VP (VP_*tot*_).

	Dilution	VP_<0.2_ (10^9^ viruses l^–1^day^–1^)	VP_*tot*_ (10^9^ viruses l^–1^day^–1^)	%VP_<0.2_	VP_*tot*_/V_*red*_
January	0.2	2.64 ± 0.73	5.78	46	1.7
	0.4	1.71 ± 0.09	4.85	35	1.4
	0.7	1.95 ± 0.49	5.09	38	1.5
	1	1.93 ± 0.19	5.08	38	1.5

	**Mean**	**2.06**	**5.20**	**39**	**1.5**

March	0.4	0.82 ± 0.18	1.78	46	1.7
	0.7	0.90 ± 0.22	1.86	48	1.8
	1	0.56 ± 0.22	1.52	37	1.5

	**Mean**	**0.76**	**1.72**	**43**	**1.7**

May	0.7	1.42 ± 0.42	14.47	10	1.0
	1	1.89 ± 0.83	14.94	13	1.0

	**Mean**	**1.66**	**14.71**	**11**	**1.0**

July	0.2	3.99 ± 1.61	11.08	36	1.3

*Errors are standard deviations (n = 3; with error propagation for the measurement using triplicate bottles). VP_tot_ was estimated according to the following equation: VP_tot_ = (1−f) × VP_red_ + VP_<0.2_, where f is the fraction of bacteria that pass through 0.2-μm filters (see Table 1). %VP_<0.2_ is the percentage of VP_<0.2_ to VP_tot_: %VP_<0.2_ = VP_<0.2_/VP_tot_× 100.*

## Discussion

Bacterial communities in the <0.2-μm filtrate were strongly enriched with LNA bacteria. In the original seawater samples, %LNA was 30–51%, whereas in the <0.2-μm filtrate, %LNA was 78–99%. This difference can be explained by selective passage of small LNA bacteria through <0.2-μm filters. Although %LNA decreased after T12 in May and July, with a concomitant increase of HNA bacterial abundance, %LNA was consistently high (>80%) during the time interval when viral abundance increased. Therefore, our data on viral dynamics in the <0.2-μm filtrate demonstrate that viral production is driven by small LNA bacteria. A high FIC (42–93% in January, March, and May), which is within the range of FIC previously reported for bulk bacterial communities (Winter et al., 2004), supports the notion that a large fraction of small LNA bacterial cells were infected with viruses (note that our FIC values are first-order estimates, given the uncertainty associated with the BS assumption and variability among dilutions, Table 2). Lytic infection of LNA bacteria by viruses has been suggested in previous studies. Based on the correlations between lytic production of viral subgroups and LNA and HNA bacterial abundance, Mojica et al. (2020) suggested that viral production is driven by both LNA and HNA bacteria in oceanic waters. Bouvier and Maurice (2011) found that 1.6% of LNA bacteria were visibly infected with viruses in the coastal Mediterranean Sea. Using this value and several assumptions, they suggested that LNA bacterial viruses account for 7% of total viral production. Our data add to this existing knowledge by directly demonstrating viral production in <0.2-μm communities dominated by LNA bacteria.

The most remarkable feature of our results is the substantial fraction (36–43%) of total viral production driven by <0.2-μm communities in January, March, and July. As bacterial abundance in the <0.2-μm filtrate represented only 8–15% of total bacterial abundance, the contribution of viral production was disproportionately high relative to bacterial abundance. Importantly, by excluding the contribution of such “hidden” viral production in <0.2-μm communities, the virus reduction assay may underestimate viral production. In fact, VP_*tot*_ was 1.3–1.7-fold higher than VP_*red*_ in January, March, and July. The results support our hypothesis that viral production in <0.2-μm communities represents a significant fraction that is missed by the virus reduction assay. Small-cell-sized bacteria of the SAR11 clade (Rappé et al., 2002), which are abundant in marine environments (Morris et al., 2002) and can be infected by viruses (Zhao et al., 2013), may be responsible for the high viral production observed in <0.2-μm communities; however, further research examining the phylogenetic compositions of the bacterial and viral communities responsible for viral production in <0.2-μm filtrates is needed to test this possibility.

We evaluated the importance of <0.2-μm communities in dissolved organic carbon (DOC) production using a model relating DOC flux (U; mmole C l^–1^ day^–1^) to bacterial carbon cell quota (Q, mmole C cell^–1^), viral production (VP, viruses l^–1^ day^–1^), and burst size (BS, viruses cell^–1^) (Motegi et al., 2009): $U=Q\times \frac{V\,P}{B\,S}.$ Using this model with assumed values of parameters including Q, VP, and BS for the <0.2-μm and >0.2-μm communities (Supplementary Text 1), the contributions of the <0.2-μm community to DOC flux were estimated to be 10% when VP_<0.2_ accounts for 40% of VP_*tot*_. This finding suggests that the <0.2-μm community can play a significant role in DOC production. We stress that the above estimation, obtained using a simple model and assumptions, is a first-order approximation. The model outputs are not generally applicable to other environments with lower contributions of VP_<0.2_ to VP_*tot*_. The model outputs are also sensitive to the parameterization of BS (Supplementary Figure 3). The relative importance of <0.2-μm communities to virus-mediated DOC cycles may differ depending on the environment and should be examined in diverse oceanographic settings in future studies.

To conclude, we found that small-cell-sized bacterial communities in <0.2-μm filtrates, which were strongly enriched in LNA bacteria, were important contributors to viral production. Viral production driven by <0.2-μm communities accounted for up to 43% of total viral production and 10% of DOM production driven by viral lysis of bacterial cells. Without consideration of viral production in <0.2-μm communities, the virus reduction assay may underestimate viral production by a factor of 1.3–1.7. This contribution was undetectable in May, suggesting that the relative importance of the <0.2-μm community depends on the season and environmental factors. It is possible that the proportion of bacteria passing through <0.2-μm filters is higher in oligotrophic oceans, where small oligotrophic bacteria are prevalent (Morris et al., 2002) than in coastal environments, leading to a greater contribution of viral production driven by <0.2-μm communities in open oceans. Furthermore, our results suggest an important area for future research concerning the role of lysogeny and chronic infection in controlling viral dynamics in <0.2-μm communities (Morris et al., 2020). Virus–bacteria interactions in <0.2-μm communities represent a significant, yet frequently overlooked, element in studies investigating the roles of viruses in marine food webs and carbon fluxes. Future research should evaluate the contributions of <0.2-μm communities in diverse oceanographic settings to improve the accuracy of viral production estimation and assessment of viral roles in biogeochemical flux regulation in the oceans.

## Data Availability Statement

The original contributions presented in the study are included in the article/Supplementary Material, further inquiries can be directed to the corresponding author.

## Author Contributions

YY designed and carried out the experiment, and also conducted flow cytometric analysis. Both authors analyzed the data and wrote the manuscript.

## Conflict of Interest

The authors declare that the research was conducted in the absence of any commercial or financial relationships that could be construed as a potential conflict of interest.

## Publisher’s Note

All claims expressed in this article are solely those of the authors and do not necessarily represent those of their affiliated organizations, or those of the publisher, the editors and the reviewers. Any product that may be evaluated in this article, or claim that may be made by its manufacturer, is not guaranteed or endorsed by the publisher.
