# Supplementary material for: Viral Production in Seawater Filtered Through 0.2-μm Pore-Size Filters: A Hidden Biogeochemical Cycle in a Neglected Realm
Source: Front Microbiol. 2021 Nov 18;12:774849. doi: 10.3389/fmicb.2021.774849 (PMC8637275; doi:10.3389/fmicb.2021.774849)
Supplement: Supplementary file 1 [file Data_Sheet_1.docx]

Supplementary Material

# Supplementary Table and Figures

## Supplementary Table

Supplementary Table 1: Summary of environmental conditions.

|  | 24 Jan  2013 | 11 Mar 2013 | 30 May 2013 | 24 July 2013 |
| --- | --- | --- | --- | --- |
| General hydrographic conditions of the bay | Water column is vertically well mixed  Pre-bloom condition | | Water column is stratified | |
| Temperature (^o^C) | 9.5 | 6.2 | 11.8 | 19.3 |
| Salinity | 33.80 | 33.59 | 33.43 | 33.41 |
| Nitrate concentration (μM) | 4.51 | 8.34 | 0.00 | 0.02 |
| NH_4_ concentration (μM) | 0.43 | 0.72 | 0.03 | 0.09 |
| PO_4_ concentration (μM) | 0.35 | 0.60 | 0.16 | 0.00 |
| Chl a concentration(μg L^-1^) | 0.77 | 0.44 | 2.00 | 3.56 |
| Viral abundance (×10^6^ particles ml^-1^) | 8.2 | 7.2 | 21 | 29 |
| Bacterial abundance (×10^6^ cells ml^-1^) | 1.1 | 0.67 | 2.1 | 3.0 |
| LNA % of total bacteria | 30 | 51 | 41 | 34 |
| Virus-to-bacteria ratio (VBR) | 7.7 | 10.8 | 10.0 | 9.7 |

## Supplementary Figures

**

**

**Supplementary Figure 1:** Changes in viral abundance during incubation of the virus reduction assays conducted in (A) January, (B) March, (C) May, and (D) July. Lines are linear regressions. Coefficient of determination (r^2^) and statistical significance are presented on each panel.

**

**

**Supplementary Figure 2:** Viral dynamics during the incubations conducted in March (A), May (B) and July (C). Bottom panels show LNA bacteria as a percentage of total bacterial abundance. For March and May, symbols indicate the dilution: open triangles, 1 dilution; closed triangles, 0.7 dilution; open circles, 0.4 dilution; closed circles, 0.2 dilution. For July, data are available for only the 0.2 dilution. Errors are standard deviations for the measurement using triplicate bottles (n = 3). Gray shaded area highlights a time interval with a significant increase in viral abundance, as indicated by asterisks. The difference in viral abundance between each time interval was tested with Student’s t test (p values <0.05 were considered significant), except for between T0 and T3, for which the difference in viral abundance was tested using the 95% confidence interval (see Materials and Method). The gray shading is not shown for T0–T3 in March, as an increase of viral abundance was observed only in the diluted sample (these data were not used for calculation of FIC and VP_< 0.2_, see Materials and Methods).





**Supplementary Figure 3:** Sensitivity of %U_< 0.2_ (values on the counter plot) to the parameterization of BS_< 0.2_ and BS_> 0.2_. See Supplementary Text 1 for explanation.

# Supplementary Text

## Supplementary Text 1

The potential contribution of viral production in the < 0.2-μm community to DOM flux was estimated using a model relating DOM flux (U; μmole C l^-1^ day^-1^) to bacterial carbon cell quota (Q, μmole C cell^-1^), viral production (VP, viruses l^-1^ day^-1^) and burst size (BS, viruses cell^-1^) (Motegi et al. 2009);

$U=Q \times VP/BS$ (Eq. 1)

Eq. 1 can be decomposed into U_> 0.2_ and U_< 0.2_, which are the DOM fluxes mediated by the > 0.2-μm and < 0.2-μm communities, respectively.

$U_{> 0.2}=Q_{> 0.2}\times{VP}_{> 0.2}/{BS}_{> 0.2}$ (Eq. 2)

$U_{< 0.2}=Q_{< 0.2}\times{VP}_{< 0.2}/{BS}_{< 0.2}$ (Eq. 3)

$U_{tot}=U_{> 0.2}+U_{< 0.2}$ (Eq. 4)

where Q_> 0.2_ and Q_< 0.2_, VP_> 0.2_ and VP_< 0.2_, and BS_> 0.2_ and BS_> 0.2_ are the Q, VP and BS values of the > 0.2-μm and < 0.2-μm communities, respectively.

Here, we consider the situation in January, March, and July, when VP_<0.2_ accounted for around 40% of VP_tot_ (VP_< 0.2_ = 0.4 × (VP_< 0.2_ + VP_> 0.2_)). Q for < 0.2-μm bacteria (Q_< 0.2_) was set to a value one-sixth of Q_> 0.2_ (Q_< 0.2_ = 1/6 × Q_> 0.2_) based on the following reasoning. Assuming that the cell size of < 0.2-μm bacteria is similar to that of the smallest SAR11-clade bacteria, which are 0.37 μm in length and 0.12 μm in diameter (Rappé et al. 2002), cell volume is approximately 0.0042 μm^3^. This volume is one-sixth of the average bacterial cell volume (0.027 μm^3^) previously determined for the dominant phylogenetic group of bacteria in Otsuchi Bay (Yokokawa & Nagata 2005). Finally, we assume that BS_> 0.2_ and BS_< 0.2_ are both 24 (BS_> 02_ = BS_< 0.2_ = 24), which is an average BS in marine communities (Parada et al. 2006). Previous studies have found no clear relationship between BS and cell size (Parada et al. 2006). Using these parameter values and Eqs. 2–4, the model output for the relative contribution of < 0.2-μm communities to total DOM flux (%U_< 0.2_ = U_< 0.2_/U_tot_ × 100) is 10%. Sensitivity analysis of %U_< 0.2_ to the parameterization of BS_< 0.2_ and BS_> 0.2_ indicates that %U_< 0.2_ exceeds 10% unless BS_< 0.2_ systematically exceeds BS_> 0.2_ (Supplementary Figure 3).

References

Motegi C, Nagata T, Miki T, Weinbauer MG, Legendre L, Rassoulzadegand F (2009) Viral control of bacterial growth efficiency in marine pelagic environments. Limnology and Oceanography 54:1901-1910

Parada V, Herndl GJ, Weinbauer MG (2006) Viral burst size of heterotrophic prokaryotes in aquatic systems. Journal of the Marine Biological Association of the United Kingdom 86:613-621

Rappé MS, Connon SA, Vergin KL, Giovannoni SJ (2002) Cultivation of the ubiquitous SAR11 marine bacterioplankton clade. Nature 418:630-633

Yokokawa T, Nagata T (2005) Growth and grazing mortality rates of phylogenetic groups of bacterioplankton in coastal marine environments. Appl Environ Microbiol 71:6799-6807
